# Women acceptance of episiotomy procedure before and after receiving educational materials: Pretest posttest study

**DOI:** 10.1016/j.eurox.2022.100161

**Published:** 2022-07-12

**Authors:** Aseel K. Haji, Suha R. Elzahrany, Rozana I. Kamal, Alanood E. Sindi, Linah K. Khairou, Rahaf M. Alahmadi, Albagir M. Hassan

**Affiliations:** a College of Medicine, Umm Al-Qura University, Makkah, Saudi Arabia; b Department of Obstetrics and Gynecology, Umm Al-Qura University, Makkah, Saudi Arabia

**Keywords:** Acceptance, Education, Episiotomy, Knowledge

## Abstract

**Objectives:**

Episiotomy is a frequently performed surgical procedure by obstetricians and midwives during vaginal birth. It is defined as a surgical incision in the perineal area through the second stage of delivery. Therefore, this study aimed to evaluate women’s acceptance toward episiotomy before and after providing education.

**Methods:**

A Quasi-experiment pretest posttest study was conducted on a total of 234 patients attending antenatal clinics in Maternity and Children hospital and Heraa General hospital in Makkah during June-August 2021. Participants were personally interviewed and provided with evidence-based information about the procedure then re-evaluated by the investigators. The statistical analysis was carried using Two Tailed Tests. Statistical significance was set on a P value of 0.05 or less.

**Results:**

Total of 234 participant fulfilling the inclusion criteria were interviewed. Females mean age is 26.2 ± 9.7 years. Exact of 115 (49.1%) women heard about episiotomy. And 79 (33.8%) correctly described it as a surgical incision. Also, 89 (38%) understood that it is not recommended for all, and 109 (46.6%) knew that anesthesia is required. The most reported source of information was internet/ social media (49%; 72), followed by friends/relatives (35.4%; 52). Before education, 112 (47.9%) would accept episiotomy if required which was significantly improved to be among 173 (73.9%) of them after receiving the educational materials.

**Conclusion:**

Due to the controversial opinions and practices of episiotomy, ensuring patients awareness and understanding is crucial. Providing correct information from trusted sources will help minimizing the chances of receiving inaccurate information from unreliable sources. Therefore, making wrong decisions, and refusing needed episiotomy. Health practitioners should be encouraged to discuss patients’ concerns and correct their misconceptions.

## Introduction

Episiotomy is a frequently performed surgical procedure by obstetricians and midwives during vaginal birth. It is defined as a surgical incision in the perineal area through the second stage of delivery [1]. Seven types of episiotomies are known. However, only three are commonly used, the midline, mediolateral and lateral [2]. This procedure aims to widen the birth canal to ease the process of delivery and avoid vaginal tears [3]. Episiotomy was first adopted in 1742 by a trained midwife and described as an emergency surgical procedure to prevent a child’s death [4]. With the advances of medicine, surgical techniques, and the emergence of anesthesia, episiotomy has become a commonly used procedure. And it has proved its great benefits; it has helped in complicated births such as those with breech position, forceps deliveries, and large newborns [5]. Episiotomy has become a routine practice and has been adopted worldwide. However, recent evidence of complications has been reported in some cases. Therefore, the new recommendation is to restrict and personalize the procedure according to each case of delivery [6]. Reported rates of episiotomy vary; it was estimated to be 14% and 75% in the United States and Canada, respectively [7]. It can cause short and long-term complications, short-term such as perineal lacerations, hemorrhage, wound site edema or infection, anal sphincter, and rectal mucosal damage. In addition, it could lead to chronic infection, anorectal dysfunction, sexual dysfunction, and pelvic organ prolapse in the long-term [8], [9]. The contradiction of experts’ opinions of episiotomy led to unnecessary fear and refusal of the procedure when advised. Thus, less provided care and poor patient satisfaction. According to Oluwasola et al. study, most pregnant women had insufficient knowledge of episiotomy and believed it is an unnecessary procedure [10], along with Odo et al. who found that most pregnant women received inaccurate episiotomy information from unreliable sources, which led to the negative attitude toward episiotomy [11] Ibrahim et al. study showed the importance of patient education during antenatal visits to increase their awareness [12]. Alexander et al. reported that antenatal education about episiotomy helped comfort women about the process of giving birth and increased the level of acceptance of the procedure when indicated [1]. Patients’ understanding and involvement in decision-making have proven to raise satisfactory levels and quality of care [13]. It is now the role of physicians to weigh the risks and benefits of episiotomy and individualize each experience of birth. Therefore, this study aimed to evaluate women’s acceptance and opinions towards episiotomy before and after receiving relevant educational materials in Makkah, Saudi Arabia.

### Conceptual framework

**Fig. 1:**
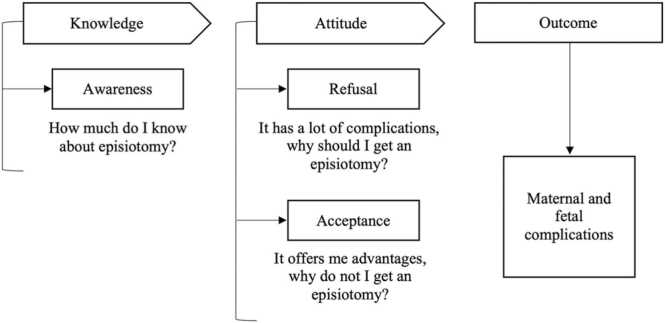
Conceptual framework of knowledge, attitude, and outcome of episiotomy.

Patients’ involvement in the process of decision-making had proven to raise satisfactory levels and quality of care [13]. Edwin et al. had advocated to adopt a patient-oriented approach to improve patients understanding [14]. Similarly, Brody et al. suggested the presence of positive relation between patients’ involvement in care plan and their attitude toward procedures and recovery [15]. Therefore, the understanding of their views and level of comprehension are crucial. Although numerous studies had acknowledged the importance of education in determining patients’ perception and behavior to any offered medical procedure, apparent deficiency in previous evidence regarding the comprehension, feelings, and behavior of pregnant women to episiotomy was found. Moreover, it is important to understand the source of the negative attitudes and misconceptions to modify these factors and raise their level of awareness (Fig. 1).

## Material and methods

### Study settings

A Quasi-experiment pretest posttest study was conducted on a total of 234 patients attending the antenatal clinics in Maternity and Children hospital and Hera General hospital in Makkah during June-August 2021. The criteria of inclusion were women in their first pregnancies and who were Makkah residents regardless to their ages and nationalities. Women who worked as physicians or refused to participate were excluded. The ethical approval was obtained from the Ethics and Research Review Committee of Umm Al-Qura University. After explaining the study aims; a verbal consent was obtained. Participants were personally interviewed about their personal information, including age, educational level, and pregnancy stage. In addition to their background about episiotomy and its source. The level of acceptance before procedure explanation was assessed using a scale from a previously published study [1]. Each participant was provided with evidence-based information about the procedure and was re-evaluated by the 5 investigators. Data were managed by Excel and analyzed using SPSS 22.

### Data analysis

Following data extraction, it was cleaned and revised, coded, then fed to statistical software IBM SPSS version 22 (SPSS, Inc. Chicago, IL). The statistical analysis was carried using Two Tailed Tests. Statistical significance was set on a P value of 0.05 or less. Regarding pregnant women awareness level of episiotomy, the frequency distribution of the correct answers was displayed. Descriptive analysis based on frequency and percent distribution was done for all variables including pregnant women socio-demographic data, pregnancy stage, educational level, their acceptance of episiotomy before and after receiving educational materials, and their source of information about episiotomy. Distribution of women acceptance of episiotomy before and after education material by their personal data and source of information was tested using Chi-Squared Test and Exact Probability Test for small frequency distributions. Also, change of females’ acceptance regarding episiotomy before and after the educational material was tested using Test of Marginal Homogeneity to assess the effect of education material on females’ decision regarding acceptance of episiotomy if required.

## Results

**Fig. 2:**
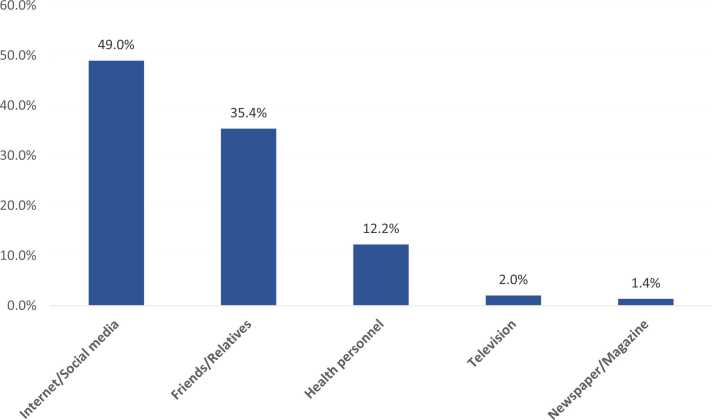
Primigravida woman source of information regarding episiotomy, Saudi Arabia.

**Fig. 3:**
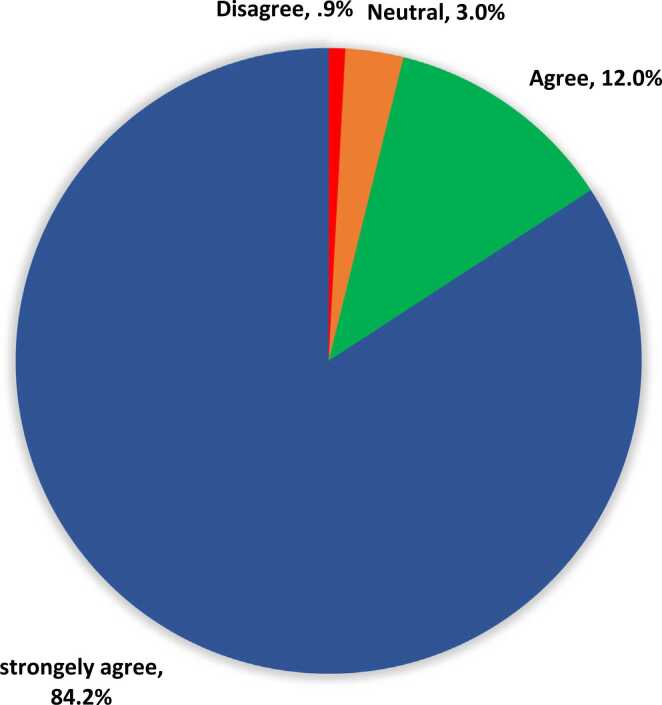
Pregnant women opinion regarding that the educational material beneficial and provided enough information.

A total of 234 pregnant women fulfilling the inclusion criteria were interviewed. Females mean age is 26.2 ± 9.7 years. Exact of 213 (91%) women were Saudis. As for educational level, 144 (61.5%) had university level of education, and 74 (31.6%) had high school education. A total of 167 (71.4%) were at their third trimester, 46 (19.7%) were at their 2nd trimester, and only 21 (9%) women were at their 1st trimester. (Table 1). Exact of 115 (49.1%) pregnant women heard about episiotomy. A total of 79 (33.8%) women correctly described episiotomy as a surgical incision. Also, 89 (38%) pregnant women said that episiotomy is not recommended for all pregnant women, and 109 (46.6%) know that anesthesia is required before episiotomy (Table 2). The most reported source of information was internet and social media (49%; 72), followed by friends and relatives (35.4%; 52), health personnel (12.2%; 18), newspaper/magazines (1.4%; 2), and television (2%; 3) (Fig. 2). Before education materials, 112 (47.9%) of the pregnant women would accept episiotomy if required which was significantly improved to be among 173 (73.9%) of them after receiving the educational materials. Also, 89 (38%) of the pregnant women reported the need of more information before the education material who were significantly decreased to only 3 (1.3%) women after the educational materials with recorded statistical significance (P = .036) (Table 3). Exact of 225 (96.2%) of the pregnant women agreed that the education material was beneficial and provided enough information while only 2 (0.9%) refused that assumption (Fig. 3). Exact of 52.7% of the women aged 25–30 years would accept episiotomy if required compared to 50% of those who aged 18–24 years and 29.6% of women aged more than 35 years with recorded statistical significance (P = .001). Also, 54.9% of women with university level of education would accept episiotomy if required versus 25% of those who had low level of education (P = .020). Additionally, 62.6% of women who previously heard about episiotomy would accept if required compared to 14.6% of those who were not sure about that procedure (P = .001) (Table 4). Exact of 80% of pregnant women aged 31–35 would accept episiotomy after education material compared to 78.2% of others aged 18–24 years and only 48.1% of those who aged more than 35 years (P = .005). Also, 76.1% of Saudi women accept episiotomy if required versus 52.4% of non-Saudi women (P = .041). Accepting episiotomy if required was also reported by 77.8% of females in their 3rd trimester compared to 61.9% of those at their 1sttrimester (P = .002). Additionally, 76.9% of women who had their information from friends/relatives would accept episiotomy if required compared to none of those who had information from magazines (P = .001) (Table 5).

**Table 1:** Personal data of sampled pregnant women, Saudi Arabia.

**Personal data**	**No**	**%**
**Age in years**		
*< 18*	13	5.6%
*18–24*	78	33.3%
*25–30*	91	38.9%
*31–35*	25	10.7%
*> 35*	27	11.5%
**Nationality**		
*Saudi*	213	91.0%
*Non-Saudi*	21	9.0%
**Level of education**		
*Less than high school*	16	6.8%
*High school*	74	31.6%
*Bachelor / above*	144	61.5%
**Pregnancy stage**		
*1st trimester*	21	9.0%
*2nd trimester*	46	19.7%
*3rd trimester*	167	71.4%

**Table 2:** Pregnant women awareness regarding episiotomy, Saudi Arabia.

**Awareness items**	**No**	**%**
**Have you heard about episiotomy before?**		
Yes	115	49.1%
No	71	30.3%
Not sure	48	20.5%
**Procedure description**		
Surgical incision	79	33.8%
Tear	40	17.1%
Operation	11	4.7%
I don't know	104	44.4%
**Is episiotomy for all women?**		
Yes	18	7.7%
No	89	38.0%
I don't know	127	54.3%
**Is anaesthesia required before episiotomy?**		
Yes	109	46.6%
No	18	7.7%
I don't know	107	45.7%

**Table 3:** The acceptance of pregnant women to episiotomy before and after education materials.

**The acceptance of pregnant women to episiotomy**	**No**	**%**	**p-value**
**The acceptance of pregnant women to episiotomy before educational material**			.036*
*I would accept and episiotomy always (Routine)*	0	0.0%	
*I would accept an episiotomy if required*	112	47.9%	
*I would not accept an episiotomy under any circumstances*	33	14.1%	
*I don’t have enough information*	89	38.0%	
**The acceptance of pregnant women to episiotomy after educational material**			
*I would accept and episiotomy always (Routine)*	14	6.0%	
*I would accept an episiotomy if required*	173	73.9%	
*I would not accept an episiotomy under any circumstances*	44	18.8%	
*I need more information*	3	1.3%	

P: Marginal homogeneity test

* P < .05 (significant)

**Table 4:** Association between demographic data and the acceptance of procedure before receiving education material.

Personal data	The acceptance of pregnant women to episiotomy before educational material						p-value
	Accept an episiotomy if required		I would not accept an episiotomy under any circumstances		I don’t have enough information		
	No	%	No	%	No	%	
**Age in years**							.001 *^,^$
*< 18*	5	38.5%	0	0.0%	8	61.5%	
*18–24*	39	50.0%	5	6.4%	34	43.6%	
*25–30*	48	52.7%	14	15.4%	29	31.9%	
*31–35*	12	48.0%	2	8.0%	11	44.0%	
*> 35*	8	29.6%	12	44.4%	7	25.9%	
**Nationality**							.108
*Saudi*	105	49.3%	27	12.7%	81	38.0%	
*Non-Saudi*	7	33.3%	6	28.6%	8	38.1%	
**Level of education**							.020 *
*Less than high school*	4	25.0%	1	6.3%	11	68.8%	
*High school*	29	39.2%	13	17.6%	32	43.2%	
*Bachelor / above*	79	54.9%	19	13.2%	46	31.9%	
**Pregnancy stage**							.139
*1st trimester*	8	38.1%	2	9.5%	11	52.4%	
*2nd trimester*	19	41.3%	4	8.7%	23	50.0%	
*3rd trimester*	85	50.9%	27	16.2%	55	32.9%	
**Have you heard about episiotomy before?**							.001*
*Yes*	72	62.6%	23	20.0%	20	17.4%	
*No*	33	46.5%	6	8.5%	32	45.1%	
*Not sure*	7	14.6%	4	8.3%	37	77.1%	
**Source of information**							.134^,^$
*Friends/Relatives*	28	53.8%	7	13.5%	17	32.7%	
*Health personnel*	6	33.3%	3	16.7%	9	50.0%	
*Newspaper/Magazine*	1	50.0%	1	50.0%	0	0.0%	
*Television*	0	0.0%	1	33.3%	2	66.7%	
*Internet/social media*	43	59.7%	14	19.4%	15	20.8%	

P: Pearson X2test.

$ Exact probability test.

* P < .05 (significant).

**Table 5:** Association between demographic data and the acceptance of procedure after receiving education material.

Personal data	The acceptance of pregnant women to episiotomy after educational material								p-value
	I would accept and episiotomy always (Routine)		I would accept an episiotomy if required		I would not accept an episiotomy under any circumstances		I need more information		
	No	%	No	%	No	%	No	%	
**Age in years**									.005*
*< 18*	2	15.4%	10	76.9%	1	7.7%	0	0.0%	
*18–24*	6	7.7%	61	78.2%	9	11.5%	2	2.6%	
*25–30*	4	4.4%	69	75.8%	17	18.7%	1	1.1%	
*31–35*	2	8.0%	20	80.0%	3	12.0%	0	0.0%	
*> 35*	0	0.0%	13	48.1%	14	51.9%	0	0.0%	
**Nationality**									.041*
*Saudi*	13	6.1%	162	76.1%	36	16.9%	2	.9%	
*Non-Saudi*	1	4.8%	11	52.4%	8	38.1%	1	4.8%	
**Level of education**									.125
*Less than high school*	1	6.3%	10	62.5%	4	25.0%	1	6.3%	
*High school*	6	8.1%	49	66.2%	17	23.0%	2	2.7%	
*Bachelor / above*	7	4.9%	114	79.2%	23	16.0%	0	0.0%	
**Pregnancy stage**									.002*
*1st trimester*	4	19.0%	13	61.9%	4	19.0%	0	0.0%	
*2nd trimester*	3	6.5%	30	65.2%	10	21.7%	3	6.5%	
*3rd trimester*	7	4.2%	130	77.8%	30	18.0%	0	0.0%	
**Have you heard about episiotomy before?**									.579
*Yes*	6	5.2%	84	73.0%	25	21.7%	0	0.0%	
*No*	4	5.6%	53	74.6%	12	16.9%	2	2.8%	
*Not sure*	4	8.3%	36	75.0%	7	14.6%	1	2.1%	
**Source of information**									.001*
*Friends/Relatives*	3	5.8%	40	76.9%	9	17.3%	0	0.0%	
*Health personnel*	3	16.7%	11	61.1%	4	22.2%	0	0.0%	
*Newspaper/Magazine*	1	50.0%	0	0.0%	1	50.0%	0	0.0%	
*Television*	0	0.0%	1	33.3%	1	33.3%	1	33.3%	
*Internet/social media*	2	2.8%	55	76.4%	15	20.8%	0	0.0%	

P: Exact probability test.

* P < 0.05 (significant).

## Discussion

Episiotomy practice is still an area of conflict among patients and physicians as well. This study showed that increasing awareness regarding the procedure improved primigravidaes' perception and therefore accepting episiotomy if indicated. When only half of the participants reported hearing about episiotomy, the majority could not describe the procedure correctly, which implies poor awareness regarding the procedure among patients and primigravidae precisely. The results align with those reported by Inyang-Eto et al. [16]. In contrast, other studies revealed that a large number of women heard about episiotomy and knew the accurate description of it [11], [12], [17].

Moreover, it might be because multiparous women in those studies since previous pregnancies and experience would contribute to gaining information and procedure familiarity compared to none in primigravidae. A significant change was observed in women's acceptance behavior toward episiotomy if required when compared between before and after the procedure explanation. While Alexander et al. reported that most women included in their study agreed to perform required episiotomy even before receiving the educational materials [1]. Age was a substantial factor affecting primigravidae acceptance of episiotomy when they are in an indicated situation both before and after receiving the intended education. Patients aged over 35 years had higher refusal rates of episiotomy regardless of any circumstances. Women at 25–30 years of age were more aware of episiotomy [18] and exhibited the maximum level of acceptance of required episiotomy compared to their peers in other age groups. The association of accepting required episiotomy with participants' level of education and knowing the procedure before were statistically significant. Patients with a higher level of education who possess proper knowledge showed further acceptance to the procedure even before providing education. However, they lost their significance after the intervention. Moreover, it was thought to be a consequence of the equal knowledge between participants after getting educated regardless of their degree or previous information. A similar significant association was reported by Abubakar et al. [19]. The mistaken thoughts and exaggerated fear might be due to the participants' unreliable sources. As most of the women said the internet, social media, friends, and relatives were their primary sources of information, as mentioned by Abubakar et al. as well [19]. While 12.2% only reported that health practitioners provided them with the information. The results disagreed with the findings of Aluwasola et al. that about one-third of those included in their study had their information provided by health care practitioners [10]. The pregnancy stage and primigravidaes' source of information did not influence their attitudes regarding the procedure before the intervention. Nevertheless, a significant change of acceptance was found after education. Owing to the equal knowledge delivered to all participants.

## Conclusion

Episiotomy is a widely performed procedure. Because of the controversial opinions and practices, patients should receive explanations about it, its indications, and benefits. Providing the correct information from trusted sources will help minimize the chances of inaccurate information from unreliable sources. Therefore, making wrong decisions, seeking inappropriate or refusing needed episiotomy. Health practitioners should be encouraged to discuss patients’ concerns and correct their misconceptions and deceptive beliefs.

## Limitations

The study was conducted in Makkah City with participant recruitment from only 2 hospitals. Further extensive studies in multiple hospitals are recommended to include more patients from various backgrounds with broader socio economic and cultural characteristics.

## Funding

This research did not receive any specific grant from funding agencies in the public, commercial, or notfor- profit sectors.

## CRediT authorship contribution statement

Aseel K. Haji conceptualized the study. Suha R. Elzahrany, Rozana I. Kamal, Alanood E. Sindi, Linah K. Khairou, Rahaf M. Alahmadi and Aseel K. Haji collected the data. Aseel K. Haji and Albagir M. Hassan analyzed it. All authors contributed to writing and reviewing the manuscript equally.

## Declaration of Competing Interest

The authors have no conflict of interest to declare.
